# Exploring Human Sperm Metabolism and Male Infertility: A Systematic Review of Genomics, Proteomics, Metabolomics, and Imaging Techniques

**DOI:** 10.3390/ijms26157544

**Published:** 2025-08-05

**Authors:** Achraf Zakaria, Idrissa Diawara, Amal Bouziyane, Noureddine Louanjli

**Affiliations:** 1 Research Laboratory of Microbiology, Infectious Diseases, Allergology and Pathogen Surveillance (LARMIAS), Mohammed VI Faculty of Medicine, Mohammed VI University of Sciences and Health (UM6SS), Casablanca 82403, Morocco; azakaria@um6ss.ma (A.Z.); idiawara@um6ss.ma (I.D.); 2 LABOMAC IVF Centers and Clinical Laboratory Medicine, Casablanca 20080, Morocco; n.louanjli@gmail.com; 3 Mohammed VI Higher Institute of Biosciences and Biotechnologies, Mohammed VI University of Sciences and Health (UM6SS), Casablanca 82403, Morocco; 4 Research Unit, Mohammed VI Center for Research and Innovation, Rabat 10112, Morocco; 5 Cheikh Khalifa International University Hospital, Casablanca 82403, Morocco; 6 Mohammed VI International University Hospital, Bouskoura 27182, Morocco; 7 IVF Center IRIFIV, Iris Clinic, Casablanca 20330, Morocco

**Keywords:** sperm metabolism, mitochondrial dysfunction, male infertility, metabolomics, proteomics, molecular biomarkers, seminal microbiome, advanced imaging, sperm bioenergetics

## Abstract

Male infertility is a multifactorial condition often associated with disruptions in sperm metabolism and mitochondrial function, yet traditional semen analysis provides limited insight into these molecular mechanisms. Understanding sperm bioenergetics and metabolic dysfunctions is crucial for improving the diagnosis and treatment of conditions such as asthenozoospermia and azoospermia. This systematic review synthesizes recent literature, focusing on advanced tools and techniques—including omics technologies, advanced imaging, spectroscopy, and functional assays—that enable comprehensive molecular assessment of sperm metabolism and development. The reviewed studies highlight the effectiveness of metabolomics, proteomics, and transcriptomics in identifying metabolic biomarkers linked to male infertility. Non-invasive imaging modalities such as Raman and magnetic resonance spectroscopy offer real-time metabolic profiling, while the seminal microbiome is increasingly recognized for its role in modulating sperm metabolic health. Despite these advances, challenges remain in clinical validation and implementation of these techniques in routine infertility diagnostics. Integrating molecular metabolic assessments with conventional semen analysis promises enhanced diagnostic precision and personalized therapeutic approaches, ultimately improving reproductive outcomes. Continued research is needed to standardize biomarkers and validate clinical utility. Furthermore, these metabolic tools hold significant potential to elucidate the underlying causes of previously misunderstood and unexplained infertility cases, offering new avenues for diagnosis and treatment.

## 1. Introduction

Human spermatozoa are highly specialized cells responsible for delivering the paternal genome to the oocyte, a process requiring substantial energy, primarily in the form of adenosine triphosphate (ATP), to maintain sperm integrity and motility [1]. ATP is generated mainly through glycolysis and oxidative phosphorylation (OXPHOS) pathways localized in distinct sperm compartments [2,3].These metabolic pathways are dynamically regulated during sperm development, maturation, capacitation, and transit through the male and female reproductive tracts [4]. Despite its critical role, sperm metabolism and bioenergetics remain underexplored, limiting advances in diagnosing and treating male infertility conditions such as asthenozoospermia and azoospermia [5].

Infertility affects approximately 8–12% of couples worldwide, with male factors contributing to nearly half of these cases [6,7].

Male infertility can result from reproductive tract blockages, hormonal imbalances, testicular dysfunction, and abnormal sperm quality or motility. Lifestyle factors such as smoking, excessive alcohol use, and obesity also negatively impact fertility. Additionally, exposure to environmental toxins can reduce sperm count and quality. These diverse factors collectively contribute to impaired male reproductive function [6].

Among male infertility disorders, sperm motility defects have been increasingly linked to mitochondrial dysfunction and impaired energy metabolism [8].

Emerging evidence also implicates the seminal microbiome in modulating sperm DNA integrity and metabolism, suggesting novel therapeutic targets [9]. Advances in omics technologies—including genomics, proteomics, and metabolomics combined with advanced analytical tools—now enable comprehensive profiling of sperm metabolic pathways and identification of molecular biomarkers associated with fertility outcomes [10,11,12].

This systematic review synthesizes recent advances in tools and techniques for evaluating human sperm metabolism and development. The objective is to critically assess methodologies that elucidate molecular mechanisms underlying infertility and improve the prediction of sperm retrieval success, particularly in azoospermia and metabolic impairments. We also aim to highlight the roles of mitochondria, oxidative stress, and the seminal microbiome, providing a comprehensive framework to inform ongoing clinical strategies and support the interpretation of emerging findings in male fertility diagnostics and therapeutics.

**Figure 1:**
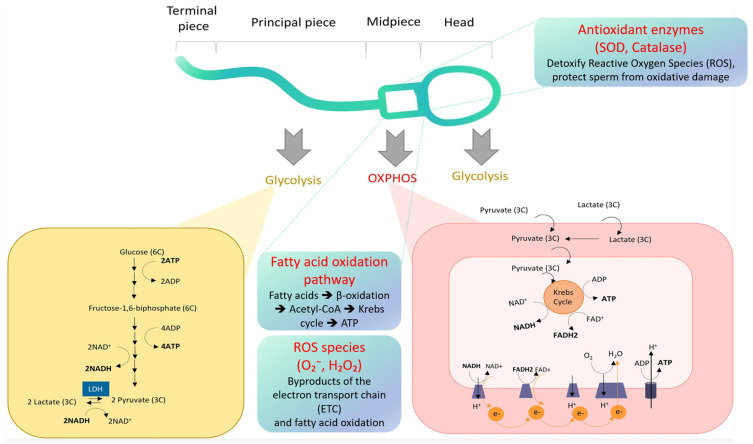
Adapted and modified from [13].

Energy metabolism in sperm cells is critical for their motility and fertilization capacity, relying primarily on glycolysis, oxidative phosphorylation (OXPHOS), and fatty acid oxidation pathways. Glycolysis occurs mainly in the sperm head and principal piece, producing ATP and metabolic intermediates, while OXPHOS and fatty acid oxidation take place in the mitochondria-rich midpiece, generating the majority of cellular ATP through the electron transport chain. However, these metabolic processes also produce reactive oxygen species (ROS), which can induce oxidative stress and impair sperm function if not adequately neutralized by antioxidant defenses. A schematic overview of these interconnected pathways and their localization within sperm cells is presented in Figure 1, providing a comprehensive framework for understanding sperm bioenergetics and oxidative balance.

## 2. Methods

### 2.1. Search Strategy

A systematic search was conducted independently by the authors using PubMed, Scopus, and Web of Science databases, covering all records published up to 15 February 2025. Search strings combined Medical Subject Headings (MeSH) and keywords linked by Boolean operators (AND, OR) to capture literature on sperm metabolism and development. The main search terms included (“sperm metabolism” OR “sperm bioenergetics”) AND (“glycolysis” OR “oxidative phosphorylation” OR “mitochondrial function” OR “mitochondrial membrane potential”) AND (“sperm motility” OR “asthenozoospermia” OR “azoospermia” OR “male infertility”) AND (“metabolomics” OR “proteomics” OR “transcriptomics” OR “antioxidants” OR “sperm capacitation” OR “microbiome”). The full search strategy is provided in Supplementary File S1.

No protocol registration (e.g., PROSPERO) was performed for this review.

### 2.2. Inclusion and Exclusion Criteria

Studies were included if they were original research articles (observational studies, clinical trials, or technical evaluations) involving human males with infertility of various etiologies, including idiopathic and metabolic-related dysfunctions affecting sperm quality.

Exclusion criteria comprised review articles, animal studies, and male infertility cases with well-established causes (e.g., varicocele, cryptorchidism, infections, genetic abnormalities, systemic diseases). The rationale for excluding animal studies was to focus on clinical relevance to human infertility.

### 2.3. Data Extraction and Synthesis

**Figure 2:**
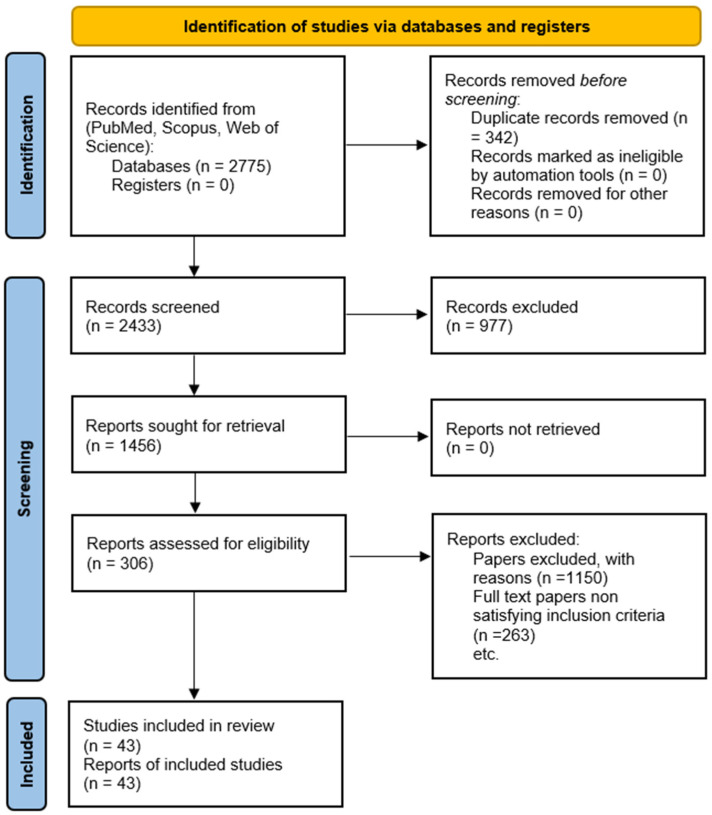
PRISMA flowchart illustrating the selection process of studies included in the systematic review.

Data on study design, sample size, diagnostic tools, and outcomes were extracted independently by two authors. Studies were grouped by analytical technique (proteomics, metabolomics, imaging, etc.) and infertility condition to facilitate synthesis. A PRISMA flowchart detailing the study selection process is provided in Figure 2, and the PRISMA checklist is included in the Supplementary File S1. The included studies are summarized and grouped by technique in Table 1A (Genomics), Table 1B (Proteomics), Table 1C (Metabolomics), and Table 1D (Imaging, Functional Assays, and Others).

## 3. Results

A total of 2775 records were identified, with 2433 remaining after duplicate removal. Screening titles and abstracts excluded 977 records, leaving 1456 for full-text review. After further exclusion of 1150 studies based on eligibility criteria, 306 texts were assessed, resulting in 43 studies being included in this review (Figure 2). Figure 2 follows the PRISMA (Preferred Reporting Items for Systematic Reviews and Meta-Analyses) guidelines.

This review synthesizes the most relevant and recent tools and techniques used to evaluate human sperm metabolism and development, focusing on their application in male infertility diagnostics and research. The included studies provide a comprehensive overview of methodologies assessing metabolic pathways, mitochondrial function, and molecular biomarkers associated with various infertility causes.

Before discussing the findings on these techniques and their clinical relevance, subsequent sections summarize current knowledge on sperm metabolism and its critical role in male fertility, providing the foundation for future research and clinical applications.

## 4. Energy Metabolism Pathways in Spermatozoa

Energy metabolism in spermatozoa depends on a series of tightly regulated biochemical reactions culminating in the production of adenosine triphosphate (ATP), the cell’s primary energy currency [51]. Two principal pathways generate ATP: glycolysis and oxidative phosphorylation (OXPHOS). Glycolysis occurs mainly in the sperm’s principal piece and head, providing a rapid but limited ATP supply without requiring oxygen [2]. This pathway yields only two net ATP molecules per glucose molecule.

OXPHOS, in contrast, takes place in the mitochondria densely packed within the sperm midpiece. This oxygen-dependent pathway is more efficient and produces the bulk of ATP needed to meet the high energy demands of sperm motility [3]. ATP generated in the midpiece must be transported along the flagellum to power movement. While diffusion has been proposed as a mechanism, its efficiency is limited by the narrow space within the flagellum, suggesting specialized transport systems might be involved, though these remain to be fully elucidated in humans and other mammals [52,53].

## 5. Metabolic Substrates and Adaptations

### 5.1. Metabolic Substrates and Transport

Sperm utilize both endogenous and exogenous carbohydrates to satisfy their metabolic needs. Seminal plasma from various mammals, including humans, contains fructose as the main metabolic substrate [54,55], along with glucose and, in some species, mannose [56]. These sugars are internalized through glucose transporters (GLUTs) with varying substrate specificities [57,58]. Additionally, sugars such as sorbitol serve as energy substrates in species including humans, rams, and mice [59,60].

Mitochondrial substrates include lactate and pyruvate, metabolized via lactate dehydrogenase (LDH) to regenerate NAD+ or enter the Krebs cycle for OXPHOS [61]. Pyruvate and lactate may originate from glycolysis or be taken up externally via monocarboxylate transporters [62,63]. Human sperm express specific LDH isoforms, enhancing mitochondrial utilization of lactate, permitting efficient respiratory substrate use [64,65]. Fatty acid β-oxidation also contributes to ATP production. Boar and bull sperm metabolize long-chain fatty acids such as oleic, palmitic, and myristic acids via mitochondrial β-oxidation [66,67]. Human sperm possess enzymatic machinery for both saturated and unsaturated fatty acid β-oxidation; inhibition of this pathway impairs motility [68]. Glycogen reserves, once thought absent in mature sperm, have been found in some species, such as dogs, where sperm can synthesize and utilize glycogen as an energy source [53,69,70].

### 5.2. Metabolic Adaptations During Maturation and Transit

Many studies use epididymal sperm to assess basal metabolic activity; however, epididymal sperm lack exposure to seminal plasma, which provides a fluid rich in energetic substrates and metabolic modulators [71]. Differences in metabolic activity between epididymal and ejaculated sperm have been observed. For example, ejaculated bull sperm rely heavily on OXPHOS for motility, whereas epididymal sperm maintain ATP levels primarily through glycolysis [71,72,73]. In mice, epididymal sperm depend mainly on glucose, but ejaculated sperm utilize a broader range of substrates, including citrate and pyruvate, through nonglycolytic pathways [74].

Sperm acquire fertilizing capacity through capacitation, a process involving extensive biochemical and physiological changes such as plasma membrane remodeling, ion fluxes, and activation of signaling cascades including cAMP-dependent pathways. Metabolic activity increases substantially during capacitation, reflecting heightened energy demands [75]. During transit through the female reproductive tract, sperm encounter variable environments with differing oxygen and substrate availability, necessitating metabolic adaptability to maintain motility and viability until fertilization [74,76,77].

## 6. Molecular Biomarkers and Oxidative Stress

Disruptions in sperm metabolism are frequently characterized by mitochondrial dysfunction and heightened oxidative stress, both pivotal in male infertility pathophysiology. Mitochondria, the powerhouse of sperm cells, are essential for ATP production necessary for motility and overall sperm function. However, excessive generation of reactive oxygen species (ROS)—a byproduct of impaired mitochondrial electron transport chains—leads to oxidative damage targeting mitochondrial membranes, proteins, and mitochondrial DNA [42]. This oxidative insult diminishes sperm motility and compromises nuclear DNA integrity, increasing the risk of mutations and fragmentation that can adversely affect fertilization and embryo development [4].

Advances in high-throughput omics technologies, including proteomics, metabolomics, and transcriptomics, have enabled the identification of specific molecular biomarkers indicative of these metabolic disturbances. Proteomic analyses reveal aberrant expression of key mitochondrial proteins and antioxidant enzymes, while metabolomic profiling uncovers altered concentrations of metabolites involved in energy metabolism and redox balance. Transcriptomic studies highlight dysregulation in gene expression pathways related to oxidative stress response and mitochondrial function [11]. Together, these molecular signatures provide valuable insight into sperm dysfunction’s biochemical underpinnings and hold promise for developing diagnostic tools and targeted therapies aimed at mitigating oxidative damage in infertile men.

## 7. Influence of the Seminal Microbiome

Recent research elucidates the seminal microbiome’s significant role—the diverse microbial communities residing within seminal fluid and associated with spermatozoa—in modulating sperm metabolic health and fertility outcomes. These microbial populations influence seminal plasma’s biochemical milieu, impacting pH, nutrient availability, and oxidative stress levels. Certain bacterial species have been implicated in either protective or detrimental effects on sperm function, with some microbes producing metabolites that exacerbate oxidative stress or trigger inflammatory responses, compromising sperm DNA integrity and metabolic efficiency [9]. The interaction between the seminal microbiome and sperm cells represents a complex, dynamic system potentially contributing to idiopathic male infertility. In healthy semen samples, the microbiota is predominantly composed of a core group of bacterial genera. The most commonly identified genera include *Lactobacillus*, *Streptococcus*, *Corynebacterium*, *Prevotella*, and *Staphylococcus*. These bacteria form the primary microbial communities in normal seminal fluid and are thought to contribute to maintaining reproductive tract health and sperm function [9]. Understanding how microbial dysbiosis alters sperm metabolism and function opens new avenues for therapeutic intervention, such as probiotics, antibiotics, or microbiome modulation strategies, to restore a healthy seminal environment. This emerging field holds promise for enhancing fertility treatments by addressing both host cellular dysfunction and microbial factors influencing reproductive health.

## 8. Advanced Techniques and Tools for Evaluating Sperm Metabolism

The evaluation of sperm metabolism and its role in male infertility has advanced considerably through diverse methodologies encompassing proteomics, metabolomics, transcriptomics, imaging, and functional assays. Agarwal et al. conducted proteomic analyses on seminal plasma from infertile men, identifying a 35-protein pathway linked to sperm dysfunction, including overexpression of membrane metallo-endopeptidase (MME) [20]. Amaral et al. characterized the sperm tail proteome, identifying 1049 proteins with prominent lipid metabolism enzymes and active mitochondrial and peroxisomal pathways, highlighting metabolic complexity essential for motility [8].

Metabolomic studies have revealed biochemical alterations in infertile populations. Boguenet et al. applied targeted metabolomics to seminal plasma from severe oligoasthenospermia patients, documenting decreased metabolites associated with membrane deterioration and energy defects [31]. Li et al. identified nine metabolites differing significantly in asthenozoospermic men, with nicotinamide levels correlating positively with sperm count and motility [78]. Zhao et al. detected disturbed metabolites implicating disrupted energy and amino acid metabolism in idiopathic asthenozoospermia [39]. Mitochondrial dysfunction emerges as a central theme linking molecular changes to impaired sperm function. Cassina et al. demonstrated mitochondrial respiratory deficits and nitro-oxidative protein modifications correlating with reduced motility [42]. Paoli et al. showed a positive correlation between mitochondrial membrane potential and sperm motility, reinforcing mitochondrial health as a fertility marker [45].

Transcriptomic and epigenetic analyses further enrich biomarker discovery. Larriba et al. profiled small RNAs in seminal extracellular vesicles from azoospermic men, identifying microRNAs discriminating azoospermia origin [16]. Li et al. reported differentially methylated regions in asthenozoospermic spermatozoa affecting genes essential for spermatogenesis and motility, suggesting epigenetic regulation contributes to metabolic dysfunction [78]. Imaging and spectroscopic techniques provide complementary functional insights. Raman microspectroscopy detects sperm DNA damage and is suitable for analyzing live sperm [46]. Magnetic resonance spectroscopy reveals motile sperm preferentially metabolize glucose, fructose, and pyruvate into lactate, highlighting increased glycolytic activity [32,38].

Oxidative stress remains critical in sperm dysfunction. Aziz et al. and Irigoyen et al. quantified ROS production, demonstrating correlations between elevated ROS and reduced mitochondrial respiratory control ratios and motility [43,48]. Proteomic analyses identified proteins differentially expressed in spermatozoa with elevated ROS, mainly related to energy metabolism and antioxidant defense [11]. Emerging research implicates the seminal microbiome in sperm health; combined 16S rRNA sequencing and metabolomics revealed Lactobacillus iners enrichment in men with high sperm DNA fragmentation, associating microbial metabolites with DNA damage and metabolic disruptions [9]. 

Integration of multi-dimensional data sets has been facilitated by bioinformatics approaches. Milardi et al. and Sharma et al. utilized pathway enrichment and network modeling to identify disrupted metabolic nodes in infertile men, accelerating biomarker discovery and therapeutic targeting{Citation}.Such systems biology approaches are crucial for translating complex molecular insights into clinical practice.

Collectively, these studies underscore sperm metabolism and its perturbations involving mitochondrial dysfunction, oxidative stress, epigenetic alterations, and microbial influences as pivotal in male infertility. The high sensitivity and specificity of these advanced methodologies promise improved diagnostic precision and personalized treatment strategies. Table 1A–D summarizes key methods and their contributions, illustrating progress toward a comprehensive assessment of sperm metabolism beyond traditional semen analysis.

## 9. Clinical Implications: Metabolic Dysfunction in Male Infertility

### 9.1. Diagnostic Implications

Metabolic biomarkers identified through omics and imaging techniques offer enhanced diagnostic precision beyond conventional semen analysis. These biomarkers can detect subtle metabolic dysfunctions contributing to infertility, enabling earlier and more accurate diagnosis.

### 9.2. Prognostic Potential

Proteomic and microRNA profiles provide prognostic information predictive of spermatogenic status and sperm retrieval success, guiding clinical decisions and personalized treatment strategies.

### 9.3. Therapeutic Strategies

Emerging interventions targeting metabolic dysfunction and seminal microbiome modulation hold promise for improving sperm function and fertility outcomes.

### 9.4. Integration with Clinical Workflows

Challenges remain in standardizing assays and integrating multi-omics data into routine clinical practice. Future research should focus on validating biomarkers in larger cohorts and developing accessible platforms for widespread clinical application.

### 9.5. Disadvantages of Current Methods and Tools to Evaluate Sperm Metabolism

Despite significant advancements, several limitations hinder the clinical translation of sperm metabolism assessment techniques. Omics approaches, while comprehensive, are often costly, time-consuming, and require specialized equipment and expertise, limiting their accessibility in routine clinical settings. Imaging techniques such as fluorescence microscopy and metabolic flux analysis provide valuable spatial and functional insights but may involve invasive sample preparation or rely on indirect metabolic indicators, potentially affecting sperm viability. Additionally, variability in sample handling, assay protocols, and data interpretation poses challenges for reproducibility and standardization across laboratories. These limitations underscore the need for developing rapid, cost-effective, and standardized tools that can reliably assess metabolism to facilitate their integration into routine infertility diagnostics.

### 9.6. Complementary Strengths of Metabolic Assessment Techniques

Different techniques offer unique strengths for studying sperm metabolism: proteomics excels in analyzing protein dynamics, metabolomics captures metabolic pathway changes, and transcriptomics reveals gene regulation. Imaging and spectroscopy provide real-time, non-invasive metabolic insights, while functional assays assess mitochondrial health and oxidative stress. Combining these complementary methods into integrated platforms will enhance biomarker discovery, improve diagnostics, and guide personalized treatments in male infertility.

## 10. Conclusions

The exploration of human sperm metabolism has greatly benefited from a variety of analytical methods and tools, ranging from traditional biochemical assays to advanced molecular and imaging technologies. This systematic review highlights the diversity and evolution of these approaches, underscoring their critical role in deepening our understanding of sperm bioenergetics and metabolic regulation. However, despite technological advances, significant challenges remain in translating these tools into standardized, clinically applicable protocols. Many methods, while powerful in research settings, lack validation for routine diagnostic use, and variability in sample preparation, assay conditions, and data interpretation limits comparability across studies.

Furthermore, the majority of current tools focus on isolated aspects of metabolism, such as glycolysis or oxidative phosphorylation, often overlooking the integrated and dynamic nature of sperm metabolic networks. There is a pressing need for comprehensive, multi-parametric platforms that can simultaneously assess multiple metabolic pathways with high sensitivity and specificity. Additionally, the accessibility and cost-effectiveness of these technologies remain barriers to widespread clinical adoption, particularly in low-resource settings.

Future research should prioritize the development and validation of standardized, user-friendly tools that can reliably assess sperm metabolism in clinical laboratories. Integration of metabolic assessments with conventional semen analysis and emerging omics data will be key to advancing personalized diagnostics and treatment strategies in male infertility. Moreover, longitudinal studies are necessary to establish the prognostic value of metabolic biomarkers and to understand how metabolic profiles evolve in response to therapeutic interventions.

Key questions for future exploration include the following: Which combinations of metabolic parameters provide the most clinically relevant information? How can emerging technologies be optimized for routine use without compromising accuracy? What are the best practices for harmonizing protocols across laboratories to ensure reproducibility? Addressing these will be essential for bridging the gap between research innovations and practical clinical applications.

In summary, while significant progress has been made in the methodological exploration of human sperm metabolism, translating these advances into clinical practice requires concerted efforts in validation, standardization, and integration. By overcoming these hurdles, we can unlock the full potential of metabolic diagnostics to improve the understanding and management of male infertility through precision medicine.

## Figures and Tables

**Table 1:** (A) Genomics studies based on techniques used in sperm metabolism and male infertility; (B) proteomics studies based on techniques used in sperm metabolism and male infertility; (C) metabolomics studies based on techniques used in sperm metabolism and male infertility; (D) imaging, functional assays, and other techniques used in sperm metabolism and male infertility.

**(A) Genomics Studies Based on Techniques Used in Sperm Metabolism and Male Infertility**					
**Methods/Tools**	**Study Aim**	**Results**	**Sample Size**	**Study Type**	**References**
MiRNA quantitative PCR panels Exosome isolation	Evaluate seminal plasma exosomal MiRNAs as markers for Azoospermia origin and sperm presence	Mir-31-5p as biomarker for azoospermia origin combined with FSH improves prediction	39	Case-control prospective study	Barceló et al., 2018 [14]
Whole-genome sequencing RNA expression analysis	Identify lncRNA mutations and expression in teratozoospermia	1166 unique mutations in Differentially Expressed lncRNAs Variants affect structure/function and MiRNA interactions	N/A *	Observational study	Kyrgiafini et al., 2023 [15]
High-throughput small RNA profiling RT-qPCR validation	Small RNA profiling in seminal extracellular vesicles for azoospermia classification	Canonical and isomiR microRNAs discriminate azoospermia origin tRNA and piRNAs less discriminatory	17	Observational case-control study	Larriba et al., 2024 [16]
Whole-genome bisulfite sequencing	Investigate DNA methylation patterns in asthenozoospermia	238 differentially methylated regions annotated to 114 genes related to spermatogenesis and motility	12	Comparative observational study	Li et al., 2022 [17]
Small RNA deep sequencing RT-qPCR validation	Study microRNA profiles in semen and testicular tissue of azoospermic men	Mir-202-3p reduced in azoospermic semen mir-370-3p elevated in azoospermia without sperm in testis	54	Observational comparative study	Wainstein et al., 2023 [18]
SOLEXA sequencing RT-qPCR	Seminal plasma miRNAs in infertile men and diagnostic value	Seven miRNAs altered miRNAs better diagnostic markers than routine parameters	457	Observational case-control study	Wang et al., 2011 [19]
**(B) Proteomics studies based on techniques used in sperm metabolism and male infertility**					
**Methods/Tools**	**Study Aim**	**Results**	**Sample size**	**Study Type**	**References**
Chemiluminescence assay Proteomic analysis via 1D gel electrophoresis In-gel digestion LC/MS-MS	Identify seminal plasma proteins involved in ROS-mediated male infertility	Membrane Metallo-Endopeptidase (MME) overexpressed; Proteins modulated in infertile groups 35-protein pathway linked to sperm dysfunction	59	Comparative observational study	Agarwal et al., 2015 [20]
LC-MS/MS Sonication and sucrose-gradient ultracentrifugation for tail fractions	Characterize human sperm tail proteome, focusing on metabolism-related proteins	1049 proteins identified; lipid metabolism enzymes prominent Mitochondrial and peroxisomal pathways active	N/A *	Experimental proteomic study	Amaral et al., 2013 [8]
Western blotting Indirect immunofluorescence	Identify and localize AMP- activated protein kinase (AMPK) in human sperm and evaluate its role in sperm motility	AMPK localized in acrosome, midpiece, tail Active AMPK higher in high motility sperm; inhibition reduces motility	N/A *	Experimental study	Calle-Guisado et al., 2017 [21]
LC-MS lipidomic analysis	Profile seminal plasma lipid composition in necrozoospermia and evaluate lipid biomarkers	Identified 1267 lipids; 20 lipids predictive for necrozoospermia Lipidomics improves diagnosis	56	Comparative observational study	Deng et al., 2024 [22]
Label-free LC-MS/MS proteomic analysis Western blot validation	Discover seminal plasma biomarkers for non-invasive differential diagnosis of Obstructive Azoospermia (OA) vs. Non-Obstructive Azoospermia (NOA)	42 proteins downregulated in NOA SCO patients Testis-specific proteins as biomarkers	30	Prospective observational proteomic study	Fietz et al., 2024 [23]
2D GEL ELECTROPHORESIS MALDI-TOF-TOF MS	Identify novel biomarkers for asthenozoospermia via sperm tail proteomic analysis	14 proteins altered in asthenozoospermia including tubulin beta 2B, glutathione S-transferase Mu 3, clusterin	N/A *	Experimental proteomic study	Hashemitabar et al., 2015 [24]
Isobaric tag labeling; LC-MS/MS Immunofluorescence western blot	Proteomic profiling of sperm in severe oligoasthenoteratozoospermia	938 proteins differentially expressed Metabolic pathways enriched YBX1 upregulated AK1 and ACO2 downregulated	N/A *	Experimental proteomic study	Liang et al., 2021 [25]
LC-MS/MS	Proteomic profile changes in spermatozoa with elevated ROS	15 proteins differentially expressed Energy metabolism and oxidative stress pathways affected	52	Prospective observational proteomic study	Sharma et al., 2013 [12]
2D-LC-MS/MS Western blot Flow cytometry Fluorometric assays	Proteomic analysis of sperm in fertilization failure after ICSI	Altered mitochondrial and proteasomal proteins in fertilization failure	17	Observational proteomic study	Massana et al., 2021 [26]
LTQ ORBITRAP mass spectrometry Protein interaction network	Identify seminal biomarkers for secondary male hypogonadism (HH)	33 proteins absent in hypogonadic patients 14 recovered after testosterone therapy	30	Observational proteomic study	Milardi et al., 2014 [10]
LC-MS/MS PROTEOMIC ANALYSIS	Identify seminal plasma proteins as biomarkers of sperm quality	20 proteins differentially expressed Biological regulation affected	N/A *	Observational proteomic study	Sharma et al., 2013 [11]
Proteomic shotgun analysis Western blot	Characterize seminal plasma proteome in primary and secondary infertility	Dysregulated proteins linked to secretion and immune response	59	Pilot observational proteomic study	Martins et al., 2020 [27]
MALDI-TOF MS TOP-DOWN PROTEIN IDENTIFICATION	Fertility-predictive model profiles of spermatozoa	High diagnostic accuracy Proteins involved in energy metabolism and sperm structure	N/A *	Experimental study	Soler et al., 2016 [28]
MALDI imaging mass spectrometry	Phospholipid expression in Sertoli cell-only syndrome (SCOS) testis	Phospholipid levels correlated with spermatogenesis Potential microTESE diagnostic tool	N/A *	Experimental study	Sulc et al., 2024 [29]
Protein quantification Incubation experiments Immunoprecipitation Mass spectrometry	Ribonuclease (RNASET2) levels in sperm and relation to motility	RNASET2 elevated in asthenozoospermia Inversely correlated with motility Interacts with AKAP4	205	Experimental observational study	Xu et al., 2018 [30]
**(C) Metabolomics studies based on techniques used in sperm metabolism and male infertility**					
**Methods/Tools**	**Study Aim**	**Results**	**Sample size**	**Study Type**	**References**
Targeted quantitative metabolomics HPLC-TANDEM MS	Assess metabolomic signatures of seminal plasma in severe Oligoasthenospermia	Decreased metabolites linked to sperm membrane deterioration and energy defects	40	Comparative observational study	Boguenet et al., 2020 [31]
Magnetic Resonance Spectroscopy (MRS)	Investigate sperm metabolism	Sperm metabolize glucose, fructose, pyruvate to lactate Motile sperm show higher glycolytic activity	97	Experimental observational study	Calvert et al., 2019 [32]
16S rRNA SEQUENCING UNTARGETED METABOLOMICS	Investigate seminal microbiome and metabolome role in high sperm DNA fragmentation index (HDFI)	Lactobacillus iners enriched in HDFI Microbial profiles linked to sperm DNA fragmentation Butanoate fermentation implicated	102	Observational case-control study	He et al., 2024 [9]
1H NMR spectroscopy (700 mhz)	Analyze seminal plasma metabolic profiles in idiopathic/male factor infertility	Distinct metabolic profiles in idiopathic infertility: Altered lysine, arginine, tyrosine, citrate, fructose	103	Observational comparative study	Jayaraman et al., 2014 [33]
Untargeted metabolomics	Correlate metabolomic profiles with semen quality in young men	Metabolites including acyl-carnitines and steroids distinguish low vs. high sperm count	2700	Observational cohort study	Olesti et al., 2023 [34]
LC-HRMS Extraction optimization	Develop steroidomics strategy for human seminal fluid	Detected 41 steroids including androgens; steroid profile stable	7	Method development and validation study	Olesti et al., 2020 [35]
1H NMR AND GC-MS 2D NMR SPECTROSCOPY	Comprehensive metabolomic characterization of human sperm cell	Identified 69 metabolites Pathways linked to sperm physiology and dysfunction	N/A *	Methodological study	Paiva et al., 2015 [36]
GC-MS	Metabolic profiling of unexplained male infertility (UMI)	Identified 44 differential metabolites Metabolic pathways altered	N/A *	Two-stage population observational study	Qiao et al., 2017 [37]
1H MRS scanning of sperm	Examine sperm molecules	Density gradient concentration (DGC) with two washes minimized contamination Metabolite peaks differed between sperm populations	20	Experimental observational study	Reynolds et al., 2017 [38]
Untargeted GC-MS metabolomics	Metabolic profiling of idiopathic asthenozoospermia sperm cells	33 metabolites identified Disturbed energy and amino acid metabolism	213	Experimental observational study	Zhao et al., 2018 [39]
HPLC-MS/MS metabolomics	Semen metabolic profiling in oligospermia patients	72–89 metabolites as potential markers Altered amino acid and ketone body metabolism	40	Comparative observational study	Zhao et al., 2024 [40]
**(D) Imaging, functional assays, and other techniques used in sperm metabolism and male infertility**					
**Methods/Tools**	**Study Aim**	**Results**	**Sample size**	**Study Type**	**References**
Fluorescence microscopy	Quantify testicular sperm apoptosis via active caspase-3 in normal and impaired spermatogenesis	Higher active caspase-3 rates in obstructive azoospermia (OA) and hypospermatogenesis Distinct localization patterns	24	Observational comparative study	Almeida et al., 2011 [41]
High-resolution respirometry Oxidative/nitrative protein detection	Analyze mitochondrial function and oxidative stress in human sperm affecting fertility	Mitochondrial dysfunction correlated with reduced motility with nitro-oxidative modifications in midpiece and head	N/A *	Experimental observational study	Cassina et al., 2015 [42]
High-resolution respirometry H_2_O_2_ measurement Antioxidant capacity assays	Assess sperm mitochondrial metabolism and ROS production as tools to complement semen analysis	Mitochondrial respiratory control ratio correlates with motility; H_2_O_2_ inversely correlated	N/A *	Experimental observational study	Irigoyen et al., 2022 [43]
ELISA Immunohistochemistry	Assess impact of seminal clusterin level on spermatogenesis and sperm retrieval in infertile men	Seminal clusterin lower in NOA and oligozoospermia Correlation with testicular expression FSH and clusterin predict retrieval	89	Observational clinical study	Fukuda et al., 2016 [44]
JC-1 fluorometric staining	Correlate sperm mitochondrial integrity with motility	Positive correlation between mitochondrial membrane potential and sperm motility	213	Experimental study	Paoli et al., 2011 [45]
Raman microspectroscopy with principal component analysis	Assessement of sperm DNA damage and biochemical features	DNA damage detected only in dry conditions Technique applicable to live sperm analysis	N/A *	Experimental methodological study	Costa et al., 2018 [46]
Raman spectroscopy Immunohistochemistry Metabolomic analysis	Identify spermatogenesis in testicular tissue	Raman spectra distinguished NOA from OA with ~90% sensitivity and 86% specificity	52	Observational diagnostic study	Liu et al., 2014 [47]
Chemiluminescence Flow cytometry with fluorescent probes	Determine cell type contributions to intracellular H_2_O_2_ and peroxynitrite production in sperm	Cell-type specific ROS production H_2_O_2_ and peroxynitrite correlated	197	Prospective observational study	Aziz et al., 2010 [48]
Fluorescence imaging; Whole-cell current clamp Pharmacological inhibitors	Progesterone-induced Ca^2+^ oscillations in human sperm	Ca^2+^ oscillations generated by CatSper channels Membrane potential modulates oscillations	20	Laboratory experimental study	Nitao et al., 2021 [49]
Assays for 21 analytes in seminal plasma ROC analysis	Evaluate seminal plasma biochemical and immunological markers in male infertility	15 markers significantly altered Monocytes/lymphocytes in NOA Platelets in asthenozoospermia	100	Observational case-control study	Vashisht et al., 2021 [50]

* N/A: The information was not available in the original study.

## Supplementary Materials

The following supporting information can be downloaded at https://www.mdpi.com/article/10.3390/ijms26157544/s1.
